# Exposure to High Salinity During Seed Development Markedly Enhances Seedling Emergence and Fitness of the Progeny of the Extreme Halophyte *Suaeda salsa*

**DOI:** 10.3389/fpls.2020.01291

**Published:** 2020-08-21

**Authors:** Jianrong Guo, Ming Du, Huaying Tian, Baoshan Wang

**Affiliations:** ^1^Shandong Provincial Key Laboratory of Plant Stress, College of Life Science, Shandong Normal University, Ji’nan, China; ^2^College of Forestry Engineering, Shandong Agriculture And Engineering University, Ji’nan, China

**Keywords:** flowering branch length, NaCl, seedling emergence, seed productivity, *Suaeda salsa*

## Abstract

Irrigation with 200 mM NaCl significantly increases vegetative and reproductive growth of the extreme halophyte *Suaeda salsa*. However, little is known about how the progeny of *S. salsa* plants grown under a continuous NaCl supply behave in terms of growth and seed set parameters. We investigated various plant growth and reproductive parameters of the progeny that germinated from seeds harvested from mother plants grown under 0 or 200 mM NaCl over three generations. Seedling emergence, plant height, stem diameter, total branch length, flowering branch length, flowering branch ratio, and seed production were all significantly enhanced in the progeny produced by mother plants grown with 200 mM NaCl compared to progeny of mother plants grown on low salinity conditions. Therefore, irrigation with 200 mM of NaCl is beneficial to seed development in the halophyte *S. salsa* and possibly contributes to population establishment in high salinity environments. Likewise, the prolonged absence of NaCl in the growth environment inhibits seed development, results in lower seed quality, and thus limits seedling growth of the progeny, thereby restricting *S. salsa* to a high salinity ecological niche.

## Introduction

Rising soil salinization is an emerging and major source of degradation of arable land. High salinity affects nearly 10% of soils and 50% of irrigated land in the world (Guo et al., 2015; Wang et al., 2015; Song et al., 2017). Furthermore, high salt environments can greatly inhibit seedling growth and yield in salt-sensitive crops (Munns et al., 2006; Kotula et al., 2015), while salt-tolerant (halophyte) plants grow well under the same conditions (Guo et al., 2018). Therefore, deciphering the mechanisms at play during salt tolerance displayed by halophytes will provide the molecular basis for a better utilization of saline-alkali soil.

Seedling emergence constitutes the most critical stage in the life cycle of plants, especially for halophytes, as it determines whether seedlings can survive in their local environment (Bajji et al., 2002). In general, high soil salinity inhibits seed germination due to the low osmotic potential created around the seed, which prevents water uptake (Welbaum et al., 1990). In addition, high concentrations of sodium and chloride ions in the soil may be toxic to seeds (Khajeh-Hosseini et al., 2003). Seedling emergence in a saline environment does, however, provide a practical and convenient assay to investigate the extent of seed sensitivity to salt.

The growth conditions mother plants experience will affect the fitness of the next generation, which can be assessed by measuring seed quality, seed size, and seedling emergence, all inter-dependent traits (Tanveer et al., 2013). For example, high nitrogen applications increased seed weight, seed vigor, and seedling vigor in cotton (*Gossypium barbadensecv*. Giza 86) (Sawan et al., 2009). In hybrid corn (*Zea mays*), fertilizing the soil with 165 kg ha^−1^ nitrogen and adequate watering of mother plants improved the germination rate and seedling vigor of the progeny (Farhadi et al., 2014). However, the effect of high salt in the environment where the mother plant grows on offspring seedling emergence remains an open question in halophytes.

The conditions experienced by the mother plants may influence other growth-related traits in the progeny, especially during reproduction. In wild oat (*Avena sativa*), mother plants infected by mycorrhizal fungi produced smaller and lighter seed, although with an elevated seed phosphorus content and a higher seed numbers in the progeny (Koide and Lu, 1992). A maternal effect can also be observed on the likelihood of flowering and on inflorescence number in the progeny of perennial ryegrass (*Lolium perenne*) (Hayward, 1967), as well as on the height of adult progeny in Aztec tobacco (*Nicotiana rustica*) (Jinks et al., 1972). Maternal effects also strongly influence progeny seed size while on the mother plant and therefore the growth parameters of the progeny over the course of their own life cycle (Dolan, 1984). It is thus critical to take into account prior exposure of the mother plant to high salt (NaCl) concentrations when investigating growth responses of the progeny collected from a halophyte.

*Suaeda salsa* is a typical annual herb extreme halophyte with succulent leaves. It grows well and produces high quality seeds when grown under high salinity conditions. At controlled condition, *S. salsa* plants could grow at the salt level with 600 mM NaCl (Song et al., 2009). While in the wild, it could complete the life cycle at the conditions containing approximate 26.5 g of salt kg^-1^ dry soil, even in the intertidal zone (Song and Xing, 2010; Wang et al., 2018). Indeed, the inclusion of 200 mM NaCl in the growth environment resulted in optimum vegetative and reproductive growth (Pang et al., 2005; Qiu et al., 2007; Qi et al., 2009; Yang et al., 2010; Li et al., 2011; Pang et al., 2011; Wang et al., 2018) and increased seed weight and seed size (Guo et al., 2018). The presence of NaCl will inhibit the germination and growth of non-halophyte seeds (Almansouri et al., 2001; Kranner et al., 2010). However, how *S. salsa* seeds, collected from mother plants exposed to high and constant salinity, respond in terms of seedling emergence and growth characteristics is unknown. Do *S. salsa* seed germinate in the absence or presence of high NaCl concentrations, and how does this influence seed quality of the progeny? Therefore, in the present study, we grew *S. salsa* over three successive generations under two growth conditions: low salt (0 mM NaCl, which we refer to as control) and high salt (200 mM NaCl). We measured seedling emergence, seedling growth, and plant productivity at each generation and compared the two salinity treatments to answer these questions.

## Materials and Methods

### Plant Material and Growth Conditions

We collected *S. salsa* seeds from the saline soils of the coastal province of Shandong, China. We stored seeds for at least 6 months in a refrigerator (<4°C), before sowing seeds in plastic buckets filled with rinsed river sand and with drainage holes at the bottom. Growth conditions were as described previously in Guo et al. (2018). We harvested seeds from individual plants irrigated without added NaCl (control) or in the presence of 200 mM NaCl and then sowed seeds in the same growth conditions as the mother plant (that is seeds harvested from a plant grown without added NaCl were sown on soil without added NaCl, while seeds from plants exposed to high salinity were sown on 200 mM NaCl). We maintained *S. salsa* plants on low or high salinity soil for three generations and harvested seeds at the end of each generation, which we used in the present study.

### *S. salsa* Seed Germination Over Three Generations

In order to determine the seed quality and the seed vigor of those harvested from multiple mother plants (exposed to 0 or 200 mM NaCl), the seed germination was performed. Four types of seeds of the three generations were treated with different concentration (0, 25, 50, 100, and 150 mM) of NaCl. The solutions used were 1/5 Hoagland dissolved with different concentration of NaCl, and 1/5 Hoagland without NaCl was considered as control. Four replicates were used for each treatment, and the seed germination percentage, germination potential, germination index, and the seed vigor index were calculated. The seed pre-processing, germination condition, observation criteria, and calculation methods were all referred to our previous study (Guo et al., 2015).

### *S. salsa* Seedling Emergence and Plant Height Over Three Generations

*S. salsa* produces two types of seeds (black and brown) on the same plant, even in the same leaf axil, in general, the former produced earlier than the latter for about 3–5 days in the same leaf axil; we harvested both types over three generations, harvested from multiple mother plants (exposed to 0 or 200 mM NaCl), and sowed the harvested seeds in rinsed river sand as described above. Before sowing, the sand was washed with complete Hoagland solution supplemented with 0, 200, or 400 mM NaCl. We recorded seedling emergence 7 DAS (days after sowing) in four replicates per NaCl concentration according to the formula: seedling emergence percentage (%) = number of emerged seedlings/number of sowed seeds×100%. We then collected plants in each pot at 30 DAS and recorded seedling height.

### Plant Phenotyping Over Three Generations

We sowed seeds harvested from plants exposed to low or high salinity under the same conditions experienced by the mother plants, as described above. At the time of 60 DAS, the growth status of plant was detected. We maintained irrigation of all plants until 105 DAS, at which time point, we measured plant height (from above sand surface) and the diameter of the main stem (at the position of 1 cm above sand surface) with a micrometer. We also recorded branch length, flower branch length and calculated the flower branch length ratio for the three generations of *S. salsa* progeny irrigated with 0 or 200 mM NaCl, when flowers started to emerge at 105 DAS.

### Seed Setting Over Three Generations

We harvested seeds from individual plants by hand at the end of the growth cycle. We therefore collected seeds from six sets of plants (three generations, two NaCl concentrations). We calculated seed yield, seed number per plant, and the weight of 1,000 seeds.

### Statistical Analysis

All results are presented as means ± SD with four to six replicates. The data were analyzed with the statistical software SPSS (ver. 17.0, SPSS Inc.) and one-way ANOVA software packages, and the univariate analysis based on the general linear model was further used. Different letters in figures indicate significant difference between the means (at *p* < 0.05) according to Duncan’s test.

## Results

### *S. salsa* Seeds Exhibit Lower Germination Indices Under Control Conditions

To investigate the effect of the growth conditions experienced by the mother plant on the seed quality and seed germination, we tested the seed germination indicators exposed to NaCl over three generations of *S. salsa* seeds harvested from mother plants grown in the presence of 0 or 200 mM NaCl (**Figures S1**–**S4**). High NaCl concentrations progressively decreased the germination percentage and the germination potential of the black seeds from mother plant grown with no NaCl, as well as with the increase of NaCl concentration, but not in the brown seeds (**Figures S1** and **S2**). With the increase of the generation, the germination index and seed vigor index were progressively decreased in the black and brown seeds from mother plant grown with no NaCl, and they were all lower than the seeds from mother plant grown with NaCl. Surprisingly, no significant defference was observed among the different geminations as to the black and brown seeds from mother plant grown with NaCl (**Figures S3** and **S4**).

### *S. salsa* Seeds Exhibit Lower Seedling Emergence Under Control Conditions

**Figure 1:**
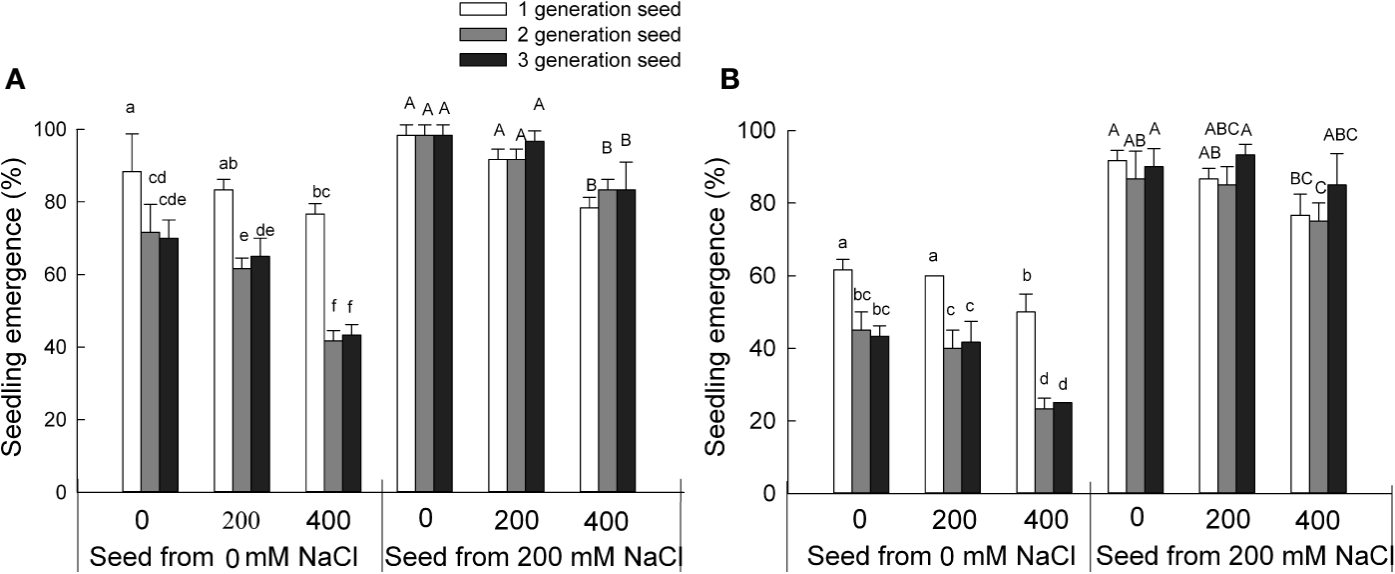
Seedling emergence rate from *S. salsa* seeds harvested from mother plants grown in 0 or 200 mM NaCl conditions when treated with 0, 200, or 400 mM NaCl. Values are means ± SD (n = 3). Different letters represent significant differences (p < 0.05) according to Duncan’s test. Black seeds **(A)**; brown seeds **(B)**.

To investigate the effect of the growth conditions experienced by the mother plant on the seed quality and, consequently, seedling growth as seeds emerge during the next generation, we tested the seedling emergence exposed to NaCl over three generations of *S. salsa* seeds harvested from mother plants grown in the presence of 0 or 200 mM NaCl (**Figure 1**). High NaCl concentrations decreased seedling emergence for seeds grown under control conditions and harvested from mother plants maintained in low salinity sand. We observed the same results for the two types of seeds (brown and black seeds) produced by *S. salsa* from mother plants grown in 0 mM NaCl. The rate of seedling emergence from black seeds was diminished between the first and third generation: Seedling emergence for third-generation seeds was 19% lower than for first-generation seeds when sown on 0 mM NaCl. Higher salt concentrations further lowered seedling emergence rates, with a 26% decrease (when grown in 200 mM NaCl) and a 46% decrease (in 400 mM NaCl) for third-generation seeds. By contrast, seedling emergence for seeds harvested from mother plants irrigated with 200 mM NaCl remained high between the first and third generation. And the major factors that affecting seedling emergence rates were the seed sources from mother plant grown in 0 or 200 mM NaCl conditions and the two types of seeds (**Table S1**). These results indicated that the initial absence of NaCl during the growth period of the mother plant markedly inhibits seedling emergence of *S. salsa* progeny regardless of their NaCl exposure. The progeny of plants grown on low salinity sand also partially lose the high salt tolerance typical for this halophyte. However, seeds harvested from mother plants grown on sand irrigated with 200 mM NaCl maintain high seed quality and seedling emergence regardless of their NaCl exposure. These results might also explain why the halophyte *S. salsa* cannot establish new populations in low salinity environments, as evidenced here by the 46% drop in seedling emergence over three generations.

### NaCl Only Reduces the Seedling Height in *S. salsa* Seeds Harvested From Plants Grown Under Control Conditions

**Figure 2:**
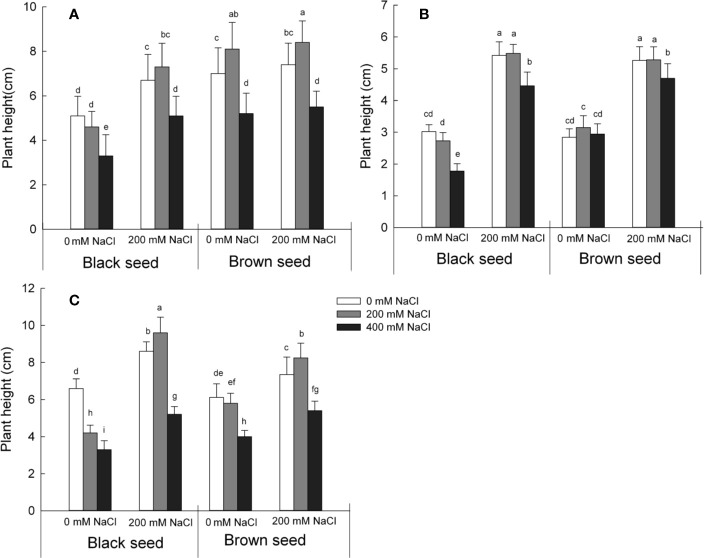
Seedlings height for plants germinated from *S. salsa* black seeds and brown seeds from mother plants grown in 0 or 200 mM NaCl conditions, when treated with 0, 200 or 400 mM NaCl. Values are means ± SD (n = 10). Different letters represent significant differences (p < 0.05) according to Duncan’s test. Seeds from the first **(A)**; second **(B)**; and third **(C)** generations.

We next measured the seedling height over three generations with seeds sown under three conditions: 0, 200, or 400 mM NaCl (**Figure 2**). High concentrations of NaCl (400 mM) inhibited plant growth as determined by seedling height relative to control conditions. By contrast, plant growth following germination of seeds harvested from mother plants exposed to 200 mM NaCl fared much better compared with those derived from mother plants grown under control conditions. Plants derived from black seeds grew taller by 31% (when grown on 0 mM NaCl), 58% (on 200 mM NaCl), and 54% (on 400 mM NaCl) when compared to plants grown under control conditions. Plants germinated from brown seeds followed a similar but weaker trend, with height increases of 6% (on 0 mM NaCl) or 4% (on 200 and 400 mM NaCl). The viability and salt tolerance of seedlings germinated from *S. salsa* seeds harvested from mother plants irrigated with 200 mM NaCl were significantly higher than those of control *S. salsa* seeds. The main factors that influenced the seedling height were the seeds harvested from mother plant grown in 0 or 200 mM NaCl and the two types of seeds that produced by *S. salsa* plant (**Table S1**). These results indicate that high salt concentrations during seed maturation promote the development of viable seed, thereby producing larger plants than seeds subjected to control conditions during maturation, especially when exposed to high salinity in subsequent generations.

### Plants Exhibit Higher Main Stem Diameter and Height in *S. salsa* Derived From Seeds Harvested From Plants Grown Under NaCl Conditions

**Figure 3:**
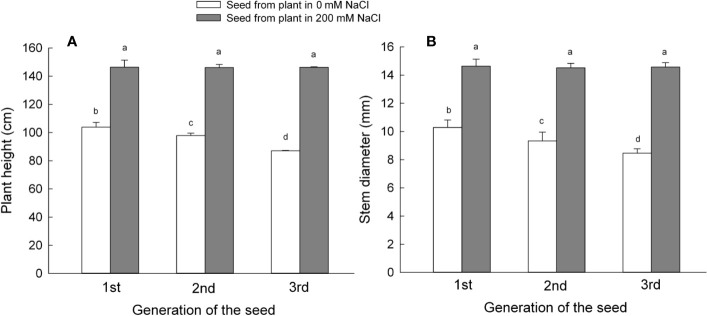
Plant growth parameters of the first, second, and third generation *S. salsa* progeny germinated from mother plants exposed to 0 or 200 mM NaCl condition, maintained in the same salinity conditions as the mother plants. Values are means ± SD (n = 7). Different letters represent significant differences (p < 0.05) according to Duncan’s test. Plant height **(A)**; stem diameter **(B)**.

To analyze the material basis of the reproductive development of *S. salsa*, we observed the growth status of plants under the treatments same as the maternal plant at 60 DAS (**Figure S5**). The formed plant of the seeds from 200 mM NaCl displayed higher biomass with a higher plant height and branch number than that from 0 mM NaCl. To determine the effects of different mother plant growth environments on later stages of progeny plant growth of *S. salsa*, we maintained irrigation of progeny, harvested from mother plants grown with 0 or 200 mM NaCl from each of the first, second, and third generations, until they started flowering (105 DAS). We then measured plant height and the diameter of the main stem (**Figure 3**). The growth environment experienced by the mother plants affected the growth of their progeny. Indeed, plants that had germinated from seeds harvested from mother plants exposed to 200 mM NaCl were taller than plants that had germinated from control seeds. Height increase ranged from 40% for first generation progeny to 68% for third generation progeny. The height of plants germinated from control seeds showed the opposite pattern, with a 6% decrease for second generation seeds and a 17% decrease for the third generation (**Figure 3A**). However, the height of plants that had germinated from seeds harvested from mother plants exposed to 200 mM NaCl remained constant over the course of the three generations.

The diameter of the plant main stem can also reflect the growth of a plant: we therefore measured the diameter of the main stem next. The different growth environments of the mother plants did affect the main stem diameter of plants germinated from seeds from the first, second, and third generations (**Figure 3B**). Progeny of plants treated with 200 mM NaCl (optimum concentration) had thicker stems compared to the progeny of mother plants maintained on low salt sand. For example, stem thickness for first generation progeny of mother plants irrigated with 200 mM NaCl was 42% greater than the diameter of matching progeny from mother plants grown with 0 mM NaCl. Main stem diameter increase rose to 55% for second generation progeny and 73% for third generation progeny; the generation was a main factor that influenced the later growth stages on the progeny plants (**Table S2**). By contrast, plant stem diameter from control plants decreased between the first and third generations, being 10% narrower during the second generation and 18% smaller during the third generation. As noted above for plant height, stem diameter from progeny continuously irrigated with 200 mM NaCl did not change over the course of our experiment, and the growth condition of the mother plant was another factor that influenced the later growth stages on the progeny plants (**Table S2**).

### Plants Exhibit Increased Total Branch Length, Flower Branch Length, and the Flower Branch Length Ratio in *S. salsa* Derived From Seeds Harvested From Plants Grown Under NaCl Conditions

**Figure 4:**
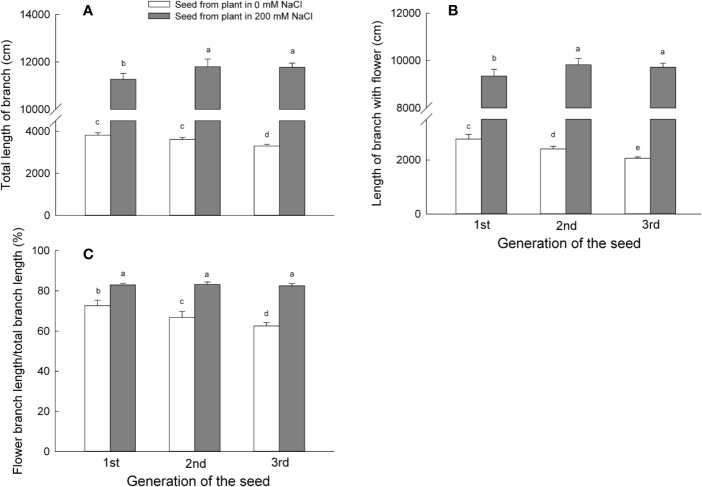
Reproductive parameters of the first, second, and third generation *S. salsa* progeny from mother plants exposed to 0 or 200 mM NaCl condition, maintained in the same salinity conditions as the mother plants. Values are means ± SD (n = 6). Different letters represent significant differences (p < 0.05) according to Duncan’s test. Length of branch per plant **(A)**; length of branch with flower per plant **(B)**; ratio of flower branch length **(C)**.

Individual plants that had germinated from seeds collected on mother plants grown in the presence of NaCl developed longer total branches when compared to plants derived from seeds harvested from mother plants grown under control conditions. Total branch length from the first generation progeny of mother plants grown in the presence of 200 mM NaCl was 3 times that of first generation progeny from those of control plants and further increased to 3.3 times (for second generation) and 3.6 times (for third generation progeny) (**Figure 4A**). It therefore appears that irrigation of mother plants with a high concentration of NaCl may be beneficial to the growth of *S. salsa* progeny, as observed here by the increase in total branch length. By contrast, the prolonged absence of NaCl irrigation during the growth cycle of mother plants impaired progeny growth, as total branch length of progeny decreased by 6% (for second generation progeny) and 14% (for third generation progeny) relative to the first generation. Again, total branch length of the progeny harvested from plants exposed constantly to 200 mM NaCl remained unchanged.

The length of flowering branches may reflect the reproductive potential of *S. salsa* plants. Plants that had germinated from seeds harvested from mother plants irrigated with 200 mM NaCl produced flowering branches longer than plants that had germinated from seeds produced by mother plants grown under control conditions: flowering branches were 3.4 times longer for first generation progeny, 4.1 times longer for second generation, and 4.7 times longer for third generation progeny, relative to control progeny (**Figure 4B**). The flowering branch length of plants constantly irrigated with 200 mM NaCl showed no differences at any generation. By contrast, plants grown under control conditions produced progressively shorter flowering branches with each generation; second generation progeny branch length reached only 86% of their first generation parents, while third generation plants had flowering branches with lengths only 74% those of the first generation plants.

We next calculated the ratio of flowering branch length as a proxy for the number of flowering buds per branch. Progeny of plants irrigated with 200 mM NaCl displayed a higher flowering branch length ratio when compared to control progeny of about 14% during the first generation, 24% during the second generation, and 31% during the third generation (**Figure 4C**). The flower bud formation in a halophyte like *S. salsa* may therefore benefit from the presence of NaCl (200 mM NaCl) during the growth period. In agreement, the flowering branch length ratio of plants maintained on 200 mM NaCl remained constant for all three generations, while it decreased in control progeny after each generation, as the ratio was 9% smaller for second generation progeny and 14% smaller for third generation progeny. The reproductive growth of the progeny plants was mainly influenced by the growth condition of mother plant, and it was also affected by the generation (**Table S2**).

### Plants Exhibit Increased Seed Production and Seed Weight of *S. salsa* Derived From Seeds Harvested From Plants Grown Under NaCl Conditions

**Figure 5:**
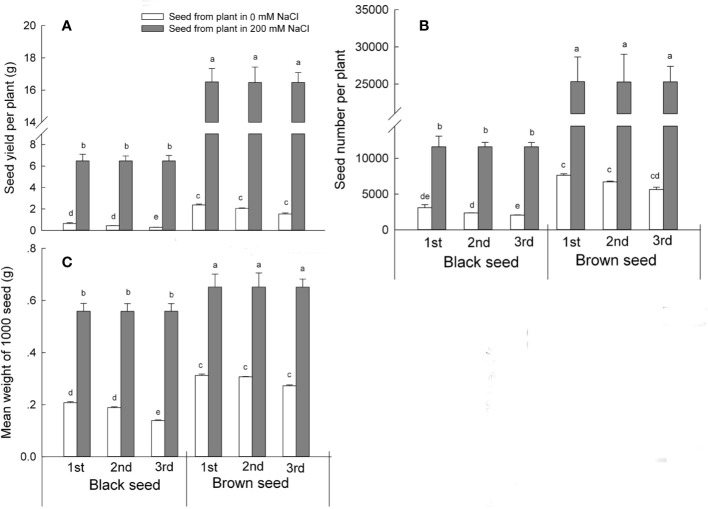
Seed parameters of first, second, and third generation *S. salsa* progeny harvested from mother plants grown in 0 or 200 mM NaCl conditions, and exposed to the same condition as their mother plants. Values are means ± SD (n = 3). Different letters represent significant differences (p < 0.05) according to Duncan’s test. Seed yield per plant **(A)**; seed number per plant **(B)**; mean 1,000-seed weight **(C)**.

We next determined seed productivity in *S. salsa* progeny harvested from mother plants irrigated with 0 or 200 mM NaCl by recording seed yield and seed number produced. Plants that had germinated from seeds harvested from mother plants grown in the presence of NaCl produced more seeds than control plants for both types of *S. salsa* seeds and for all three generations. Black seed yield was 10.2, 14.4, and 22.6 times that of matching control plants of the first, second, and third generations, respectively. Brown seed yield was about half that of black seeds, but still well above that of control plants, reaching a yield 6.9, 8, and 10.5 times that measured over the first, second, and third generations in plants grown under control conditions (**Figure 5A**). The growth condition with the mother plants of NaCl maintained the high seed productivity than those grow with no NaCl (**Table S3**). Seed number per plant followed the same trend, as the number of black seeds was 3.7, 4.9, and 5.6 times higher, and the number of brown seeds 3.3, 3.8, and 4.4 times higher than the seed number produced by plants grown under control conditions over the first, second, and third generations, respectively (**Figure 5B**). These results suggest that the presence of NaCl during plant development improved overall plant growth, resulting in higher *S. salsa* seed quality and seed yield in the progeny.

Consistent with a positive effect arising from exposure to high salinity, the progeny of *S. salsa* plants grown in the presence of NaCl formed larger seeds than control plants, as evidenced by the mean weight of 1,000 seeds (a more accurate method than weighing individual seeds). The mean-mass of 1,000 black seeds was 2.7, 2.95, and 4 times higher than in plants grown under control conditions, while the mean-mass of 1,000 brown seeds was 2.1, 2.1, and 2.4 times higher when harvested from first, second, or third generation progeny, respectively. Interestingly, black seed weight gradually and consistently decreased in *S. salsa* plants always grown on low salinity soil, with a reduction in 1,000 black seed mass of 10% for second generation and 34% for third generation progeny when compared to first generation progeny (**Figure 5C**). Brown seed weight was not affected by low salinity conditions. The prolonged relaxation of high salinity growth conditions may therefore affect *S. salsa* plant growth, seed development, and progeny seed weight. During the seed formation and seed development processes, generation was one of the factors that affected the seed quality of *S. salsa* when plants grow with no NaCl.

## Discussion

Seed quality (a collective term covering seed germination, seed size, and seedling vigor) contribute to crop yield and may influence seed germination and seedling emergence at the beginning of the following generation. Ambient temperature, light, water supply, and soil nutrient levels all constitute the growth environment experienced by the mother plant during reproduction and seed setting (Roach and Wulff, 1987). These factors will affect the performance of the progeny by limiting or promoting growth of the mother plant (Rengasamy, 2006). For instance, in hybrid sweet pepper (*Capsicum annuum* L. cv. Hazera’ 1195), seeds formed at lower temperatures (during winter) displayed a higher seedling emergence rate than seeds formed at higher temperatures (i.e., in the summer) (Xu and Kafkafi, 2003). However, *Arabidopsis thaliana* seeds that form on the mother plant in a cold environment germinate more slowly than those formed in a warm maternal environment (Blödner et al., 2007). This offers a stark contrast to Sheepgrass (*Leymus chinensis*), for which seed germination decreases with a rise in temperature and reduces flowering stalks (Gao et al., 2012). Exposure to drought during seed setting will affect seed quality and seed yield. For example, in rapeseed (*Brassica napus* L.), drought applied during sexual reproduction drastically reduces seed yield (Ahmadi and Bahrani, 2009). Similarly, water stress imposed during the blooming stage in fennel (*Nigella sativa*) and Psyllium (*Plantago ovata*) resulted in lower seed yield (Bannayan et al., 2008). Water deficit during flowering of Indian pea (*Lathyrus sativus* L.) also significantly reduced the emergence of progeny seedlings (Gusmao et al., 2012). Furthermore, the seed germination and seedling growth were also related to the utilization of the stored materials in seeds (Zhao et al., 2018a), which came from the mother plant during the seed formation.

Soil salinization is a major limiting factor for economic development of agriculture and forestry, especially in arid and semi-arid regions (McWilliam, 1986). The salinity of the environment in which maternal plants grow may affect the quality of the seeds they produce. Salt-tolerant varieties of Carolina Iris (*Iris hexagona*) produce seeds that germinate better into faster-growing seedlings when mother plants are exposed to high salinity compared to seeds produced by plants grown under lower salinity (Van Zandt and Mopper, 2004).

In this study, seed quality of the halophyte *S. salsa* was markedly affected by the level of salinity experience by the maternal plants during their growth period. Compared with seeds produced by mother plants grown on low salinity sand, both black and brown seeds produced by mother plants irrigated with 200 mM NaCl displayed higher seed germination percentage (**Figure S1**), seed vigor index (**Figure S4**), seedling emergence rates (**Figure 1**), and plant height (**Figure 2**), even when challenged in an environment lacking NaCl. We had shown previously that seeds produced by mother plants grown in the presence of 200 mM NaCl were larger and had higher protein and lipid content than seeds produced by mother plants grown on low salinity conditions (Guo et al., 2015); the grown condition of mother plant had a major effect on the seed quality of *S. salsa* (**Table S1**) and may provide an explanation for the better emergence parameters of the progeny seeds of plants grown under high salinity conditions.

Our results further suggest that some NaCl supplied during the course of the reproductive cycle of the halophyte *S. salsa* ameliorates seed development, while low salinity may inhibit seed development, reduce seed quality, and reduce seedling emergence rates (Song et al., 2016). Our results suggest that the prolonged absence of salt during halophyte growth, especially during the reproductive stage, will severely inhibit seed development, which in turn is likely to severely limit population establishment in a low salinity environments. However, routine exposure to high NaCl may support high seed performance when mother plants grow in high salinity conditions (such as 200 mM NaCl) and may provide an ecological advantage when attempting to establish or maintain a population in high salinity environments.

In addition to seed germination, seedling growth and plant growth and reproduction are also influenced by the environment experienced by the mother plants. In wild oat, the progeny produced much lighter seeds when the mother plant was infected by mycorrhizal fungi, although seed phosphorus content and seed number increased in parallel (Koide and Lu, 1992). Maternal effects also affect flowering time and inflorescence number in perennial ryegrass (*Lolium perenne*) (Hayward, 1967) and the height of adult plants in Aztec tobacco (Jinks et al., 1972).

In the present study, the progeny of *S. salsa* harvested from mother plants irrigated with 200 mM NaCl formed longer branches that had more flowers than the progeny of plants grown under control conditions (**Figure 4**), and produced larger seeds (**Figure 5C**). Therefore, one possible avenue where the maternal effect may influence plant growth is through the modulation of seed size (Dolan, 1984). For example, seed size was positively associated with reproductive yield in wild radish (*Raphanus raphanistrum*) (Stanton, 1984). The relationship between seed size and seed yield may also depend on the surrounding environment. The presence of 200 mM NaCl in the irrigation solution supplied to mother plants promoted flower bud formation during the reproductive stage of *S. salsa* progeny (**Table S2**) and may possibly be related to phytohormone content and higher nutrient supply early during seedling and plant growth (Roach and Wulff, 1987; Guo et al., 2020a).

Furthermore, any maternal effect reaches far beyond seed development (Stanton, 1984) and plays a pivotal role in the establishment of natural populations. We noticed that irrigation with high salt concentrations during plant growth all the way to seed setting produced healthy *S. salsa* seeds that germinated well on high salinity sand, whereas seeds harvested from plants maintained on low salinity conditions did not fare as well (Zhou et al., 2016). We therefore tested the consequences of lack of salt exposure to the progeny harvested from mother plants irrigated with no NaCl over three consecutive generations and compared the results to those of progeny of plants continuously exposed to high salinity (**Table S3**). Seed yield and seed number remained high for all three generations when *S. salsa* plants were grown in the presence of 200 mM NaCl; the progeny also produced more seeds when compared to the progeny of plants grown under control conditions (**Figure 5**). Seeds harvested from *S. salsa* plants always grown in the presence 200 mM NaCl maintained a constant 1,000-seed mass and high seed quality, as evidenced by their bigger seeds. By contrast, black seeds collected from plants not exposed to NaCl exhibited a gradually reduced 1,000-seed mass after each generation, with a reduction of 10% (second generation) and 34% (third generation), compared to the first generation progeny (**Figure 5C**). Since high concentrations of NaCl during seed development was beneficial to the growth of the mother plants, and thus to seed quality, the absence of salt may result in the opposite effect. Higher seed yield in high salinity conditions may rely on the induction of male reproductive organ development in *S. salsa* plants treated with NaCl (Guo et al., 2019; Guo et al., 2020b), and may also reflect increased seedling growth when treated with an appropriate concentration of NaCl (Lu et al., 2002; Lu et al., 2003; Song et al., 2008; Qi et al., 2009; Guo and Wang, 2014; Shao et al., 2014; Song and Wang, 2015; Zhou et al., 2016).

The increased vegetative and reproductive growth processes of *S. salsa* progeny plants under salt condition are ultimately inseparable from the photosynthesis of plants. Previous results showed that the photosynthesis in the leaves of *S. salsa* was enhanced when plants treated with 200 mM NaCl for a short time, such as 7 or 14 days, even at the condition of 400 mM NaCl (Lu et al., 2002; Lu et al., 2003), along with the enhanced plant biomass (**Figure S5**). When *S. salsa* plants treated with NaCl for a long period, such as the whole life cycle, an increased seed yield and seed number was obtained, and the photosynthetic efficiency in its leaves can maintain a high stable level (Guo et al., 2020c). And the enhanced reproduction when treated with NaCl was benefited from the increased accumulation of starch in the ovules and thus increased the seed size and seed development of *S. salsa* (Guo et al., 2020c). Undoubtedly, the most fundamental material source of reproductive development is from photosynthesis products of leaves, and a very small part of photosynthesis can be carried out in the petals at early developmental stage of *S. salsa* (Guo et al., 2020c). The increased photosynthetic efficiency in the leaves and flowers of *S. salsa* when treated with NaCl was ultimately inseparable from a higher chlorophyll contents both in the leaves and in the flowers, despite of the increased accumulation Na^+^ and Cl^-^ in the leaves and flowers of *S. salsa* when plants grow with NaCl. Interestingly, the biomass and the reproduction of *S. salsa* was not inhibited when grow with NaCl, but instead improved, even much salt ions was accumulated in the flowers and seeds (Guo et al., 2018; Zhao et al., 2018b). And a result of increased seed number (**Figure 5B**) and seed vigor were obtained in *S. salsa* treated with optimal concentration of NaCl (Guo et al., 2015; Guo et al., 2018).

Plant size is a strong indicator of the reproductive success. Larger plants have a relatively higher fertility or a higher vigor in their progeny (Stanton, 1984). Plant height, stem diameter, and total branch length of the progeny that had germinated from seeds collected from mother plants grown in 200 mM NaCl (**Figure 3**) were significantly higher than those of mother plants grown under control conditions. Reproductive parameters, including flowering branch length and the ratio of flowering branch in the progeny, were similarly higher in these plants. Our results strongly indicate that continuous exposure to high salinity promotes seedling growth and improves reproductive parameters, whereas omitting NaCl continuously negatively and gradually affects the reproductive growth process of the progeny, as measured by plant height and flowering branch length. Our observations are consistent with the hypothesis that the higher seed yield of larger plants may be related to their greater number of reproductive organs (de Jong and Klinkhamer, 1989). Similarly, in *Digitalis purpurea*, larger maternal plants exhibited better reproductive growth, improved progeny quality, and larger seed sets (Sletvold, 2002). And the difference in seed set might be positively associated with the metabolites content in it (Li and Song, 2019). As in the present study, prolonged exposure to high salinity soils contribute to higher seedling size and seed yield, and quality, both prerequisites for population establishment in high salinity environments. By contrast, the prolonged absence of NaCl exposure during growth of the mother plant inhibited seed development and diminished seedling quality and development of the corresponding progeny.

## Conclusions

This study constitutes the first investigation of the effects associated with the continuous absence or presence of an optimum concentration of NaCl during vegetative and reproductive growth of the progeny of the extreme halophyte *S. salsa*. We measured higher parameters in the progeny of mother plants irrigated with 200 mM NaCl over the course of all three generations, including seedling emergence rate, plant height, main stem diameter, total branch length, flowering branch length, the flowering branch ratio, and seed set, therefore covering the entire lifecycle of the plants from seed to seed. These results suggest that 200 mM NaCl supplied during *S. salsa* growth promotes healthy seed development in this halophyte and contributes to a higher potential for seedling emergence and plant development in the progeny. High salinity exposure during seed development produces high quality seeds that can begin to establish new populations in high salinity environments. By contrast, the prolonged absence of NaCl inhibits seed quality and seedling development of the halophyte *S. salsa*, thereby restricting its ecological niche to high salinity environments and preventing the establishment of new populations in a low salinity environment.

## Data Availability Statement

The original contributions presented in the study are included in the article/**Supplementary Material**; further inquiries can be directed to the corresponding author.

## Author Contributions

JG and BW conceived the original project and designed the experiments. JG and MD performed most of the experiments. MD and HT performed the statistical analysis. JG and BW wrote the article. All authors contributed to the article and approved the submitted version.

## Conflict of Interest

The authors declare that the research was conducted in the absence of any commercial or financial relationships that could be construed as a potential conflict of interest.

## Supplementary Material

Figure S1 Germination percentage from *S. salsa* seeds harvested from mother plants grown in 0 or 200 mM NaCl conditions, when treated with 0, 25, 50, 100, or 150 mM NaCl. Values are means ± SD (n = 4). Different letters represent significant differences (p < 0.05) according to Duncan’s test. Black seeds (A); brown seeds (B).

Figure S2 Germination potential from *S. salsa* seeds harvested from mother plants grown in 0 or 200 mM NaCl conditions, when treated with 0, 25, 50, 100 or 150 mM NaCl. Values are means ± SD (n = 4). Different letters represent significant differences (p < 0.05) according to Duncan’s test. Black seeds (A); brown seeds (B).

Figure S3 Germination index from *S. salsa* seeds harvested from mother plants grown in 0 or 200 mM NaCl conditions, when treated with 0, 25, 50, 100 or 150 mM NaCl. Values are means ± SD (n = 4). Different letters represent significant differences (p < 0.05) according to Duncan’s test. Black seeds (A); brown seeds (B).

Figure S4 Seed vigor index from *S. salsa* seeds harvested from mother plants grown in 0 or 200 mM NaCl conditions, when treated with 0, 25, 50, 100 or 150 mM NaCl. Values are means ± SD (n = 4). Different letters represent significant differences (p < 0.05) according to Duncan’s test. Black seeds (A); brown seeds (B).

Figure S5 Photos of *S. salsa* plants generated from seeds of mother plants grown in 0 or 200 mM NaCl conditions, and exposed to the same condition as their mother plants at 60 DAS. 0 mM NaCl (A); 200 mM NaCl (B). Bar, 5 cm.

Table S1 Multivariate analysis of variance in the seedling emergence and seedling height of *S. salsa* seeds, those were harvested from mother plants grown in 0 or 200 mM NaCl conditions, when treated with 0, 200, or 400 mM NaCl.

Table S2 Multivariate analysis of variance in the growth parameters of *S. salsa* plants, those were generated from mother plants grown in 0 or 200 mM NaCl conditions, and treated with 0 or 200 that same as the mother plants.

Table S3 Multivariate analysis of variance in the seed parameters of *S. salsa* plants, those were generated from mother plants grown in 0 or 200 mM NaCl conditions, and treated with 0 or 200 that same as the mother plants.
