# Supplementary figures and images for: Exposure to High Salinity During Seed Development Markedly Enhances Seedling Emergence and Fitness of the Progeny of the Extreme Halophyte Suaeda salsa
Source: Front Plant Sci. 2020 Aug 21;11:1291. doi: 10.3389/fpls.2020.01291 (PMC7472538; doi:10.3389/fpls.2020.01291)

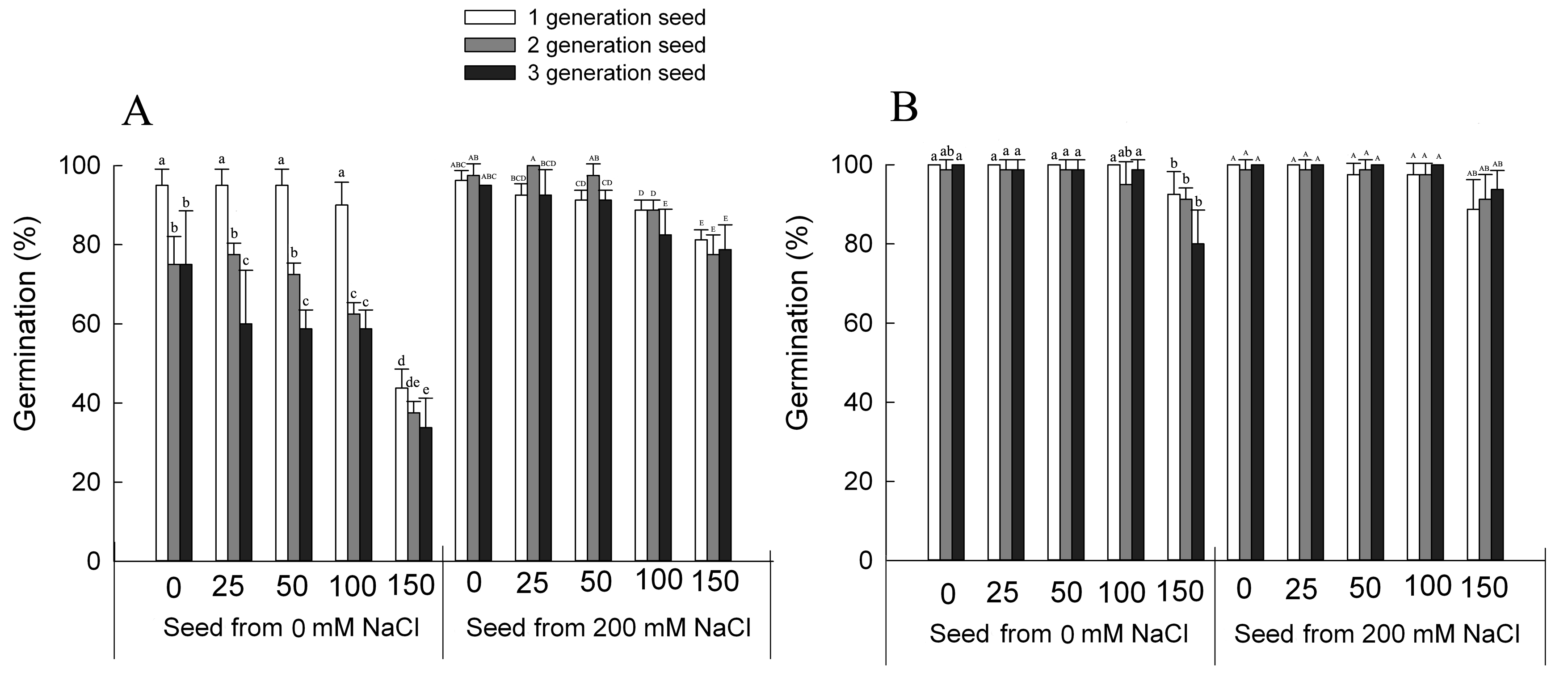

Supplement: Figure S1 — Germination percentage from S. salsa seeds harvested from mother plants grown in 0 or 200 mM NaCl conditions, when treated with 0, 25, 50, 100, or 150 mM NaCl. Values are means ± SD (n = 4). Different letters represent significant differences (p < 0.05) according to Duncan’s test. Black seeds (A); brown seeds (B). [file Image_1.tif]

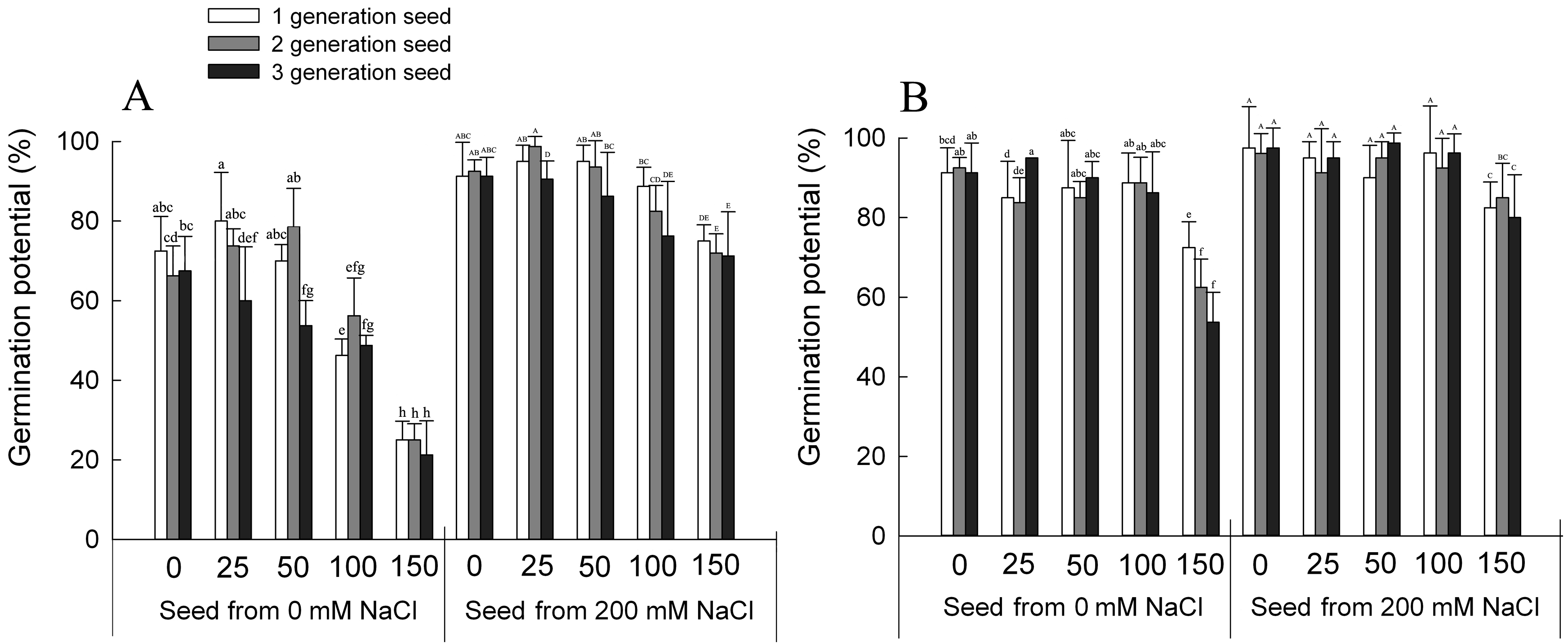

Supplement: Figure S2 — Germination potential from S. salsa seeds harvested from mother plants grown in 0 or 200 mM NaCl conditions, when treated with 0, 25, 50, 100 or 150 mM NaCl. Values are means ± SD (n = 4). Different letters represent significant differences (p < 0.05) according to Duncan’s test. Black seeds (A); brown seeds (B). [file Image_2.tif]

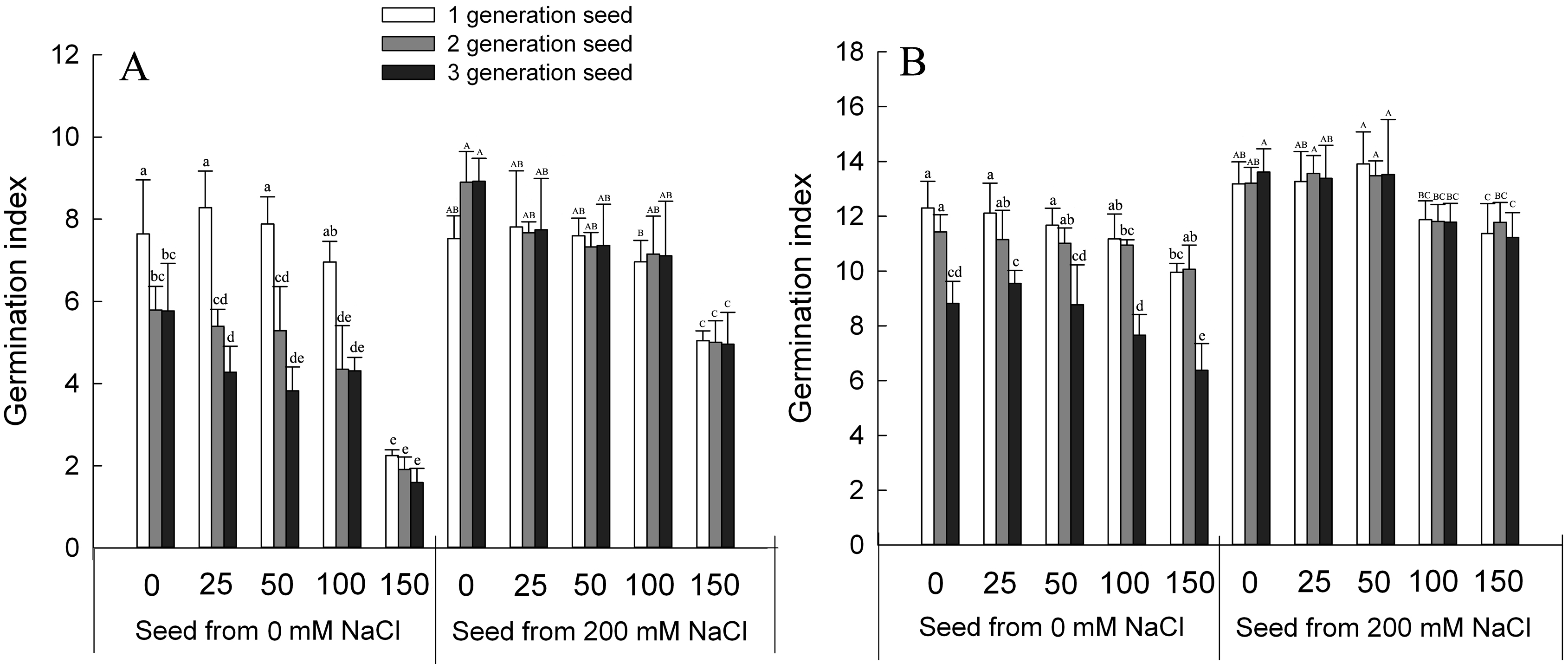

Supplement: Figure S3 — Germination index from S. salsa seeds harvested from mother plants grown in 0 or 200 mM NaCl conditions, when treated with 0, 25, 50, 100 or 150 mM NaCl. Values are means ± SD (n = 4). Different letters represent significant differences (p < 0.05) according to Duncan’s test. Black seeds (A); brown seeds (B). [file Image_3.tif]

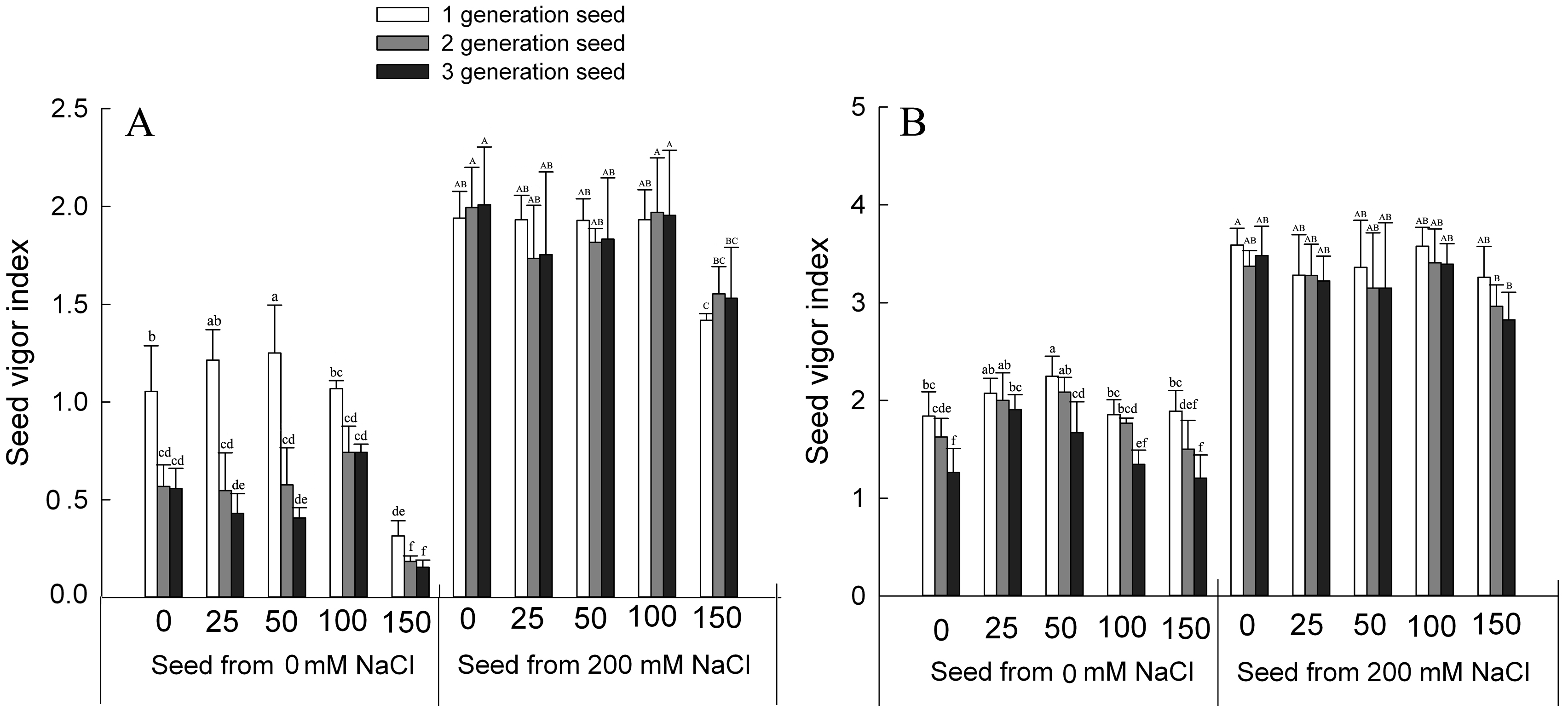

Supplement: Figure S4 — Seed vigor index from S. salsa seeds harvested from mother plants grown in 0 or 200 mM NaCl conditions, when treated with 0, 25, 50, 100 or 150 mM NaCl. Values are means ± SD (n = 4). Different letters represent significant differences (p < 0.05) according to Duncan’s test. Black seeds (A); brown seeds (B). [file Image_4.tif]

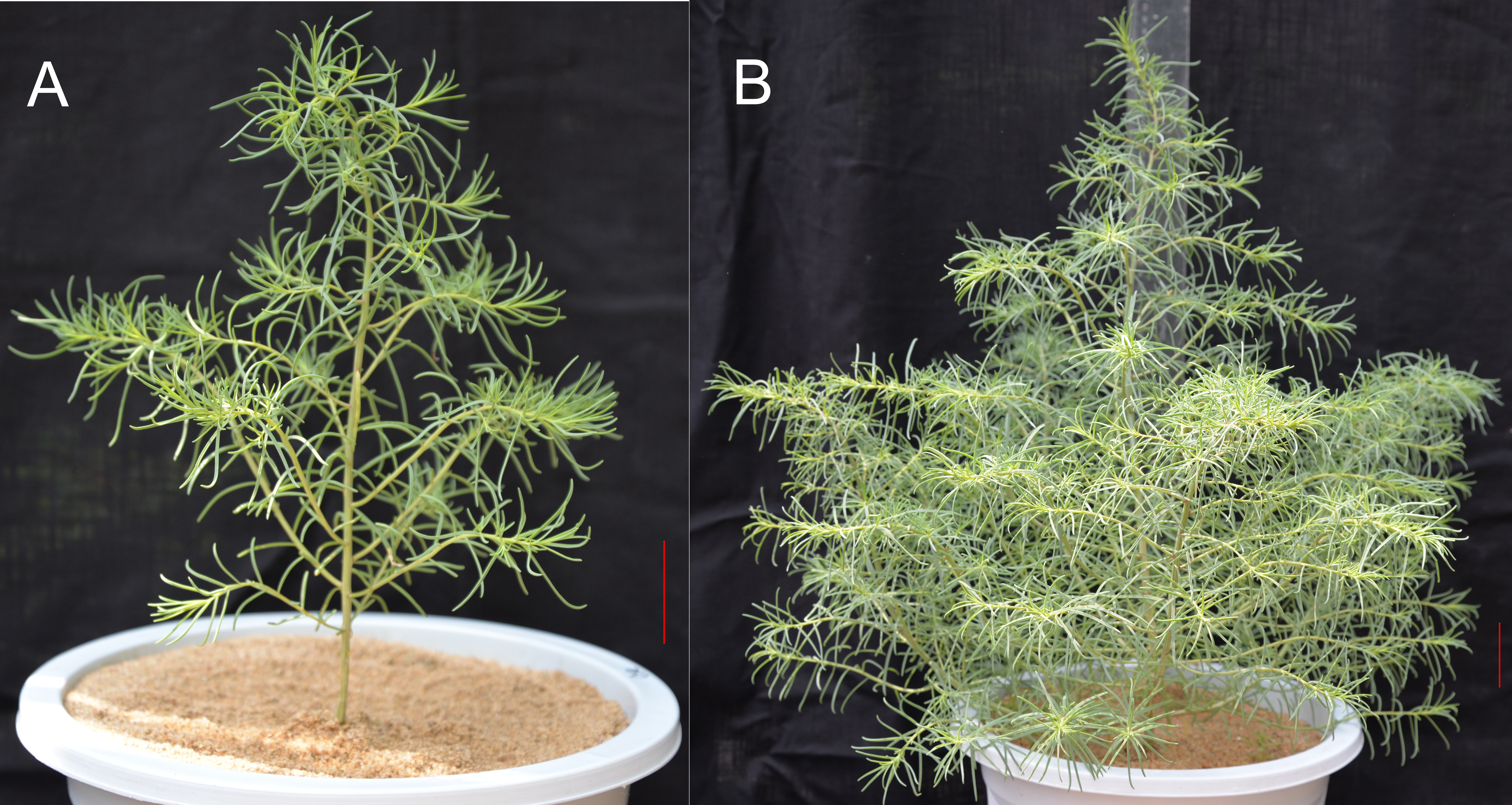

Supplement: Figure S5 — Photos of S. salsa plants generated from seeds of mother plants grown in 0 or 200 mM NaCl conditions, and exposed to the same condition as their mother plants at 60 DAS. 0 mM NaCl (A); 200 mM NaCl (B). Bar, 5 cm. [file Image_5.tif]
