# Supplementary material for: Exposure to High Salinity During Seed Development Markedly Enhances Seedling Emergence and Fitness of the Progeny of the Extreme Halophyte Suaeda salsa
Source: Front Plant Sci. 2020 Aug 21;11:1291. doi: 10.3389/fpls.2020.01291 (PMC7472538; doi:10.3389/fpls.2020.01291)
Supplement: Table S1 — Multivariate analysis of variance in the seedling emergence and seedling height of S. salsa seeds, those were harvested from mother plants grown in 0 or 200 mM NaCl conditions, when treated with 0, 200, or 400 mM NaCl. [file Table_1.docx]

| **Dependent variable** | **Factors** | **Significant** |
| --- | --- | --- |
| Seedling emergence | Mother plant | 0.00 |
|  | Seed type | 0.00 |
|  | Generation | 0.00 |
|  | NaCl concentration | 0.00 |
|  | Mother plant*Seed type | 0.00 |
|  | Mother plant*generation | 0.00 |
|  | Mother plant*NaCl concentration | 0.014 |
|  | Seed type*generation | 0.494 |
|  | Seed type*NaCl concentration | 0.031 |
|  | Generation* NaCl concentration | 0.152 |
|  | Mother plant*Seed type* generation | 0.184 |
|  | Mother plant*Seed type* NaCl concentration | 0.989 |
|  | Mother plant*generation* NaCl concentration | 0.005 |
|  | Seed type*generation* NaCl concentration | 0.767 |
|  | Mother plant*Seed type*generation* NaCl concentration | 0.835 |
| Seedling height | Mother plant | 0.00 |
|  | Seed type | 0.00 |
|  | Generation | 0.00 |
|  | NaCl concentration | 0.00 |
|  | Mother plant*Seed type | 0.00 |
|  | Mother plant*generation | 0.00 |
|  | Mother plant*NaCl concentration | 0.00 |
|  | Seed type*generation | 0.00 |
|  | Seed type*NaCl concentration | 0.00 |
|  | Generation* NaCl concentration | 0.00 |
|  | Mother plant*Seed type* generation | 0.003 |
|  | Mother plant*Seed type* NaCl concentration | 0.001 |
|  | Mother plant*generation* NaCl concentration | 0.00 |
|  | Seed type*generation* NaCl concentration | 0.00 |
|  | Mother plant*Seed type*generation* NaCl concentration | 0.044 |

Table S1 Multivariate analysis of variance in the seedling emergence and seedling height of *S. salsa* seeds, those were harvested from mother plants grown in 0 or 200 mM NaCl conditions, when treated with 0, 200, or 400 mM NaCl.
