# Supplementary material for: Exposure to High Salinity During Seed Development Markedly Enhances Seedling Emergence and Fitness of the Progeny of the Extreme Halophyte Suaeda salsa
Source: Front Plant Sci. 2020 Aug 21;11:1291. doi: 10.3389/fpls.2020.01291 (PMC7472538; doi:10.3389/fpls.2020.01291)
Supplement: Table S2 — Multivariate analysis of variance in the growth parameters of S. salsa plants, those were generated from mother plants grown in 0 or 200 mM NaCl conditions, and treated with 0 or 200 that same as the mother plants. [file Table_2.docx]

| **Dependent variable** | **Factors** | **Significant** |
| --- | --- | --- |
| Stem height | Treatment | 0.00 |
|  | Generation | 0.00 |
|  | Treatment*generation | 0.00 |
| Stem diameter | Treatment | 0.00 |
|  | Generation | 0.00 |
|  | Treatment*generation | 0.00 |
| Breach length | Treatment | 0.00 |
|  | Generation | 0.074 |
|  | Treatment*generation | 0.00 |
| Flower breach length | Treatment | 0.00 |
|  | Generation | 0.021 |
|  | Treatment*generation | 000 |
| Ratio of flower breach length | Treatment | 0.00 |
|  | Generation | 0.00 |
|  | Treatment*generation | 0.00 |

Table S2 Multivariate analysis of variance in the growth parameters of *S. salsa* plants, those were generated from mother plants grown in 0 or 200 mM NaCl conditions, and treated with 0 or 200 that same as the mother plants.
