# Supplementary material for: Exposure to High Salinity During Seed Development Markedly Enhances Seedling Emergence and Fitness of the Progeny of the Extreme Halophyte Suaeda salsa
Source: Front Plant Sci. 2020 Aug 21;11:1291. doi: 10.3389/fpls.2020.01291 (PMC7472538; doi:10.3389/fpls.2020.01291)
Supplement: Table S3 — Multivariate analysis of variance in the seed parameters of S. salsa plants, those were generated from mother plants grown in 0 or 200 mM NaCl conditions, and treated with 0 or 200 that same as the mother plants. [file Table_3.docx]

| **Dependent variable** | **Factors** | **Significant** |
| --- | --- | --- |
| Seed yield | Mother plant | 0.00 |
|  | Seed type | 0.00 |
|  | Generation | 0.00 |
|  | Mother plant* Seed type | 0.00 |
|  | Mother plant* generation | 0.00 |
|  | Seed type*generation | 0.00 |
|  | Mother plant* Seed type*generation | 0.00 |
| Seed number | Mother plant | 0.00 |
|  | Seed type | 0.00 |
|  | Generation | 0.358 |
|  | Mother plant* Seed type | 0.00 |
|  | Mother plant* generation | 0.657 |
|  | Seed type*generation | 0.796 |
|  | Mother plant* Seed type*generation | 0.993 |
| Seed mean weight | Mother plant | 0.00 |
|  | Seed type | 0.00 |
|  | Generation | 0.056 |

Table S3 Multivariate analysis of variance in the seed parameters of *S. salsa* plants, those were generated from mother plants grown in 0 or 200 mM NaCl conditions, and treated with 0 or 200 that same as the mother plants.
